# Supplementary figures and images for: Searching for a common host: parasitoids of Lema daturaphila on Datura stramonium in Central Mexico
Source: PeerJ. 2025 Feb 3;13:e18675. doi: 10.7717/peerj.18675 (PMC11801200; doi:10.7717/peerj.18675)

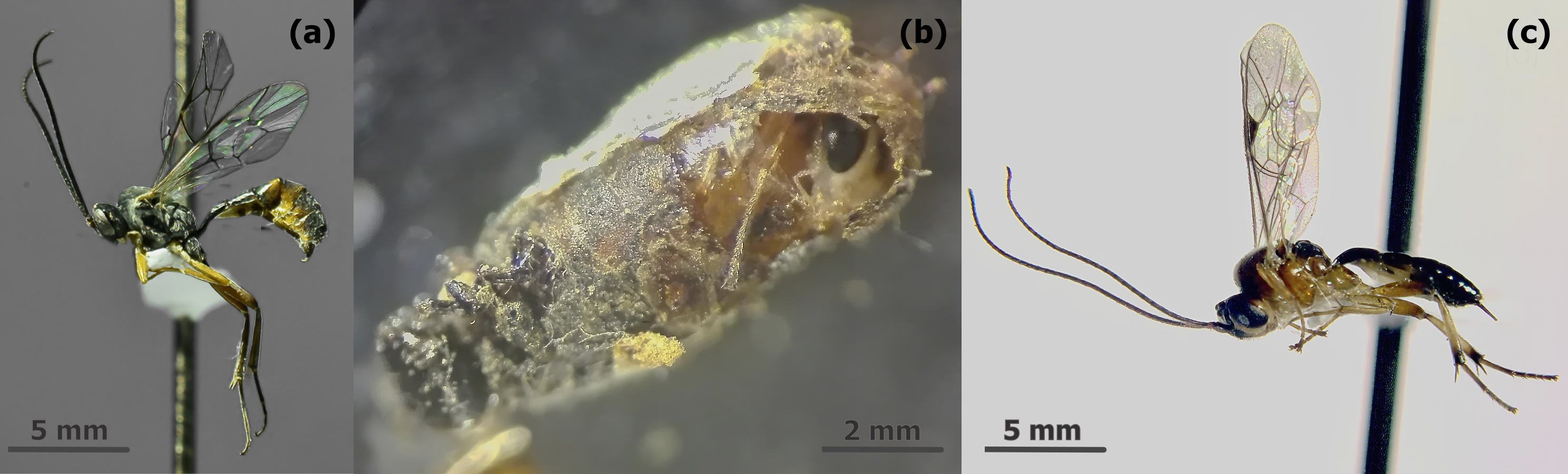

Supplement: Supplemental Information 1 — Ichneoumonidae parasitoid found in Pedregal population (a), hyperparasitoid emerging from a pupae cocoon (b), and lateral view of Mesochorus sp. (c). [file peerj-13-18675-s001.png]
