# Supplementary material for: Searching for a common host: parasitoids of Lema daturaphila on Datura stramonium in Central Mexico
Source: PeerJ. 2025 Feb 3;13:e18675. doi: 10.7717/peerj.18675 (PMC11801200; doi:10.7717/peerj.18675)
Supplement: Supplemental Information 2 — Geographic location and average climatic conditions per year of the sampled localities. P = annual precipitation (mm) and T = annual temperature (°C). [file peerj-13-18675-s002.docx]

| **Population** | **State** | **Latitude** | **Longitude** | **Altitude (m. a. s. l.)** | **Precipitation (mm)** | **Temperature (°C)** |
| --- | --- | --- | --- | --- | --- | --- |
| Bernal | Querétaro | 20.7300 | -99.9300 | 2081 | 465.5 | 17.3 |
| Dolores | Querétaro | 20.3512 | -100.105 | 2136 | 616.5 | 18.5 |
| Pedregal | Mexico City | 19.3100 | -99.1900 | 2320 | 816.9 | 17.5 |
| Requena | Hidalgo | 19.9200 | -99.3300 | 2119 | 642.5 | 16.7 |
| San Martín | Puebla | 19.2704 | -98.4279 | 2259 | 760.6 | 15.6 |
| Teotihuacán | Mexico | 19.6800 | -98.8300 | 2290 | 616.6 | 15.9 |
| Texcoco | Mexico | 19.4997 | -98.8990 | 2244 | 508.3 | 15.6 |
| Tlaxiaca | Hidalgo | 20.1102 | -98.8902 | 2367 | 403.2 | 15.3 |
| Toluca | Mexico | 19.1296 | -99.4493 | 2640 | 828.9 | 12.9 |
| Tzintzuntzán | Michoacán | 19.6300 | -101.570 | 2051 | 669.7 | 16.4 |
| Valsequillo | Puebla | 18.9163 | -98.1089 | 2078 | 668.3 | 16.2 |
