# Supplementary material for: Searching for a common host: parasitoids of Lema daturaphila on Datura stramonium in Central Mexico
Source: PeerJ. 2025 Feb 3;13:e18675. doi: 10.7717/peerj.18675 (PMC11801200; doi:10.7717/peerj.18675)
Supplement: Supplemental Information 3 — Number of egg clutches, eggs, and larvae collected in each locality during both years. The last row shows the data summed per year. [file peerj-13-18675-s003.docx]

| **Population** | **2018** | | | **2019** | | |
| --- | --- | --- | --- | --- | --- | --- |
|  | **Egg clutches** | **Eggs** | **Larvae** | **Egg clutches** | **Eggs** | **Larvae** |
| Bernal | 17 | 207 | 50 | 38 | 729 | 51 |
| Dolores | - | - | - | 34 | 732 | 77 |
| Pedregal | 30 | 648 | 5 | 40 | 878 | 90 |
| Requena | 18 | 227 | 26 | 50 | 890 | 14 |
| San Martín | - | - | - | 8 | 109 | 65 |
| Teotihuacán | 25 | 516 | 7 | 48 | 1,061 | 152 |
| Texcoco | 32 | 610 | 3 | 55 | 1,170 | 153 |
| Tlaxiaca | 19 | 452 | 72 | 37 | 658 | 130 |
| Toluca | 10 | 212 | 31 | 35 | 603 | 75 |
| Tzintzuntzán | 12 | 226 | 0 | 30 | 1,018 | 21 |
| Valsequillo | 32 | 554 | 3 | 36 | 635 | 10 |
| **Total** | 195 | 3,652 | 197 | 411 | 8,483 | 838 |
