# Supplementary material for: Searching for a common host: parasitoids of Lema daturaphila on Datura stramonium in Central Mexico
Source: PeerJ. 2025 Feb 3;13:e18675. doi: 10.7717/peerj.18675 (PMC11801200; doi:10.7717/peerj.18675)
Supplement: Supplemental Information 4 — Estimated values for the number of Lema daturaphila eggs per clutch based on population, year, and their interaction. The estimates were obtained using a negative binomial generalized linear model, with Bernal as the reference population. The model explains 19.23% of the variance, and the interaction between population and year is indicated by an asterisk in the first column. [file peerj-13-18675-s004.docx]

|  | **Estimate** | **Std. Error** | **Z value** | **Pr(>\|z\|)** |
| --- | --- | --- | --- | --- |
| Intercept | 2.49 | 0.105 | 23.69 | <2e-16*** |
| Dolores | 0.11 | 0.093 | 1.23 | 0.216 |
| Pedregal | 0.57 | 0.127 | 4.49 | 6.85e-06*** |
| Requena | 0.03 | 0.146 | 0.23 | 0.810 |
| San Martín | -0.34 | 0.163 | -2.09 | 0.036* |
| Teotihuacán | 0.52 | 0.131 | 4.00 | 6.15e-05*** |
| Texcoco | 0.44 | 0.126 | 3.53 | 0.0004*** |
| Tlaxiaca | 0.66 | 0.137 | 4.86 | 1.16e-06*** |
| Toluca | 0.55 | 0.162 | 3.40 | 0.0006*** |
| Tzintzuntzán | 0.43 | 0.156 | 2.78 | 0.005** |
| Valsequillo | 0.35 | 0.127 | 2.75 | 0.005*** |
| 2019 | 0.45 | 0.123 | 3.67 | 0.0002*** |
| Dolores*2019 | NA | NA | NA | NA |
| Pedregal*2019 | -0.43 | 0.155 | -2.81 | 0.004** |
| Requena*2019 | -0.10 | 0.170 | -0.647 | 0.517 |
| San Martín*2019 | NA | NA | NA | NA |
| Teotihuacán*2019 | -0.38 | 0.157 | -2.45 | 0.014* |
| Texcoco*2019 | -0.34 | 0.151 | -2.26 | 0.023* |
| Tlaxiaca*2019 | -0.74 | 0.166 | -4.49 | 7.13e-06*** |
| Toluca*2019 | -0.66 | 0.188 | -3.15 | 0.0004*** |
| Tzintzuntzán*2019 | 0.13 | 0.182 | 0.73 | 0.461 |
| Valsequillo*2019 | -0.43 | 0.158 | -2.75 | 0.005** |
| **Null deviance:** 775.40 on 605 degrees of freedom | | | | |
| **Residual deviance:** 626.28 on 586 degrees of freedom | | | | |
| **AIC:** 4213.8 | | | | |
