# Supplementary material for: Searching for a common host: parasitoids of Lema daturaphila on Datura stramonium in Central Mexico
Source: PeerJ. 2025 Feb 3;13:e18675. doi: 10.7717/peerj.18675 (PMC11801200; doi:10.7717/peerj.18675)
Supplement: Supplemental Information 5 — Mean, median, and standard deviation in the number of eggs per clutch of Lema daturaphila in all sampled populations. [file peerj-13-18675-s005.docx]

| **Population** | **2018** | | | **2019** | | |
| --- | --- | --- | --- | --- | --- | --- |
|  | **Mean** | **Median** | **Standard deviation** | **Mean** | **Median** | **Standard deviation** |
| Bernal | 12.17 | 11 | 6.58 | 19.18 | 20 | 7.45 |
| Dolores | - | - | - | 21.52 | 21.5 | 7.47 |
| Pedregal | 21.6 | 20.5 | 8.61 | 21.95 | 22 | 7.34 |
| Requena | 12.61 | 11.5 | 6.69 | 17.8 | 17.5 | 7.58 |
| San Martín | - | - | - | 13.62 | 9.5 | 11.04 |
| Teotihuacan | 20.64 | 22 | 7.58 | 22.10 | 21.5 | 8.29 |
| Texcoco | 19.06 | 17 | 7.55 | 21.27 | 22 | 7.08 |
| Tlaxiaca | 23.78 | 20 | 12.03 | 17.78 | 17 | 8.29 |
| Toluca | 21.2 | 21.5 | 8.58 | 17.22 | 15 | 6.61 |
| Tzintzuntzan | 18.83 | 15.5 | 11.74 | 33.93 | 34 | 9.35 |
| Valsequillo | 17.31 | 16 | 7.53 | 17.63 | 15.5 | 7.04 |
