# Supplementary material for: Searching for a common host: parasitoids of Lema daturaphila on Datura stramonium in Central Mexico
Source: PeerJ. 2025 Feb 3;13:e18675. doi: 10.7717/peerj.18675 (PMC11801200; doi:10.7717/peerj.18675)
Supplement: Supplemental Information 6 — Estimated values for the parasitism of Lema daturaphila clutches based on population, year, and their interaction. The estimates were obtained using a binomial generalized linear model, with Bernal as the reference population. The model explains 56.6% of the variance, and the interaction between population and year is indicated by an asterisk in the first column. [file peerj-13-18675-s006.docx]

|  | **Estimate** | **Std. Error** | **Z value** | **Pr(>\|z\|)** |
| --- | --- | --- | --- | --- |
| Intercept | -1.54 | 0.636 | -2.42 | 0.015* |
| Dolores | 19.20 | 1744.53 | 0.011 | 0.99 |
| Pedregal | 0.84 | 0.744 | 1.13 | 0.25 |
| Requena | 3.14 | 0.897 | 3.51 | 0.0004*** |
| San Martín | 19.05 | 1744.53 | 0.011 | 0.99 |
| Teotihuacán | -18.02 | 2150.8 | -0.008 | 0.99 |
| Texcoco | 1.66 | 0.728 | 2.287 | 0.0221* |
| Tlaxiaca | 1.001 | 0.794 | 1.261 | 0.20 |
| Toluca | -18.02 | 3400.71 | -0.005 | 0.99 |
| Tzintzuntzán | 3.93 | 1.223 | 3.22 | 0.001** |
| Valsequillo | 3.22 | 0.801 | 4.028 | 5.63e-05*** |
| 2019 | -18.02 | 1744.53 | -0.01 | 0.99 |
| Dolores*2019 | NA | NA | NA | NA |
| Pedregal*2019 | 38.28 | 2436.1 | 0.016 | 0.987 |
| Requena*2019 | 19.59 | 1744.5 | 0.011 | 0.991 |
| San Martín*2019 | NA | NA | NA | NA |
| Teotihuacán*2019 | 18.02 | 3174.6 | 0.006 | 0.99 |
| Texcoco*2019 | 19.07 | 1744.5 | 0.011 | 0.99 |
| Tlaxiaca*2019 | 17.10 | 1744.5 | 0.010 | 0.99 |
| Toluca*2019 | 35.22 | 3822.07 | 0.009 | 0.99 |
| Tzintzuntzán*2019 | 35.19 | 2626.47 | 0.013 | 0.98 |
| Valsequillo*2019 | 35.90 | 2501.17 | 0.014 | 0.98 |
| **Null deviance:** 839.43 on 605 degrees of freedom | | | | |
| **Residual deviance:** 364.09 on 586 degrees of freedom | | | | |
| **AIC:** 404.09 | | | | |
