# Supplementary material for: Searching for a common host: parasitoids of Lema daturaphila on Datura stramonium in Central Mexico
Source: PeerJ. 2025 Feb 3;13:e18675. doi: 10.7717/peerj.18675 (PMC11801200; doi:10.7717/peerj.18675)
Supplement: Supplemental Information 7 — Estimates were calculated with a binomial generalized model. In 2019, the clutch size of Lema daturaphila had a significant effect on the probability of being parasitized (GLM, p = 0.0254). [file peerj-13-18675-s007.docx]

|  | **2018** | | | |
| --- | --- | --- | --- | --- |
|  | **Estimate** | **Std. Error** | **Z value** | **Pr(>\|z\|)** |
| Intercept | 0.0954 | 0.33457 | 0.285 | 0.775 |
| Eggs per clutch | -0.0133 | 0.01622 | -0.824 | 0.410 |
| **Null deviance:** 269.17 on 194 degrees of freedom | | | | |
| **Residual deviance:** 268.49 on 193 degrees of freedom | | | |  |
| **AIC:** 272.49 | | | |  |
|  | **2019** | | | |
|  | **Estimate** | **Std. Error** | **Z value** | **Pr(>\|z\|)** |
| Intercept | -0.3647 | 0.25815 | -1.413 | 1.1577 |
| Eggs per clutch | 0.02608 | 0.01167 | 2.235 | 0.0254* |
| **Null deviance:** 566.78 on 410 degrees of freedom | | | | |
| **Residual deviance:** 561.66 on 409 degrees of freedom | | | | |
| **AIC:** 565.66 | | | | |
