# Supplementary material for: Searching for a common host: parasitoids of Lema daturaphila on Datura stramonium in Central Mexico
Source: PeerJ. 2025 Feb 3;13:e18675. doi: 10.7717/peerj.18675 (PMC11801200; doi:10.7717/peerj.18675)
Supplement: Supplemental Information 8 — Estimated values about the number of emerged Emersonella lemae. The number of eggs per clutch and the population were used as predictors. Estimates were calculated with a negative binomial generalized linear model and back-transformed to the original measure scale. For 2018, populations were statistically different from Bernal, the reference population. The model explains 38.05% of the variance for 2018 and 66.87% of the variance for 2019. [file peerj-13-18675-s008.docx]

|  | **2018** | | | |
| --- | --- | --- | --- | --- |
|  | **Estimate** | **Std. Error** | **Z value** | **Pr(>\|z\|)** |
| Intercept | 0.851 | 4.708e-01 | -0.343 | 0.731 |
| Eggs per clutch | 1.043 | 1.527e-02 | 2.802 | 0.00508** |
| Pedregal | 1.578 | 5.445e-01 | 0.838 | 0.40206 |
| Requena | 4.669 | 5.730e-01 | 2.689 | 0.00717** |
| Teotihuacán | 9.081 | 2.736e+05 | 0.000 | 0.99 |
| Texcoco | 2.918 | 5.263e-01 | 2.036 | 0.04178* |
| Tlaxiaca | 1.276 | 6.035e-01 | 0.405 | 0.68 |
| Toluca | 8.812 | 4.310e+05 | 0.000 | 0.99 |
| Tzintzuntzán | 5.635 | 6.370e-01 | 2.714 | 0.00665* |
| Valsequillo | 4.590 | 5.199e-01 | 2.931 | 0.00338** |
| **Null deviance:** 266.58 on 194 degrees of freedom | | | | |
| **Residual deviance:** 165.14 on 185 degrees of freedom | | | | |
| **AIC:** 843.22 | | | | |
|  | **2019** | | | |
|  | **Estimate** | **Std. Error** | **Z value** | **Pr(>\|z\|)** |
| Intercept | 5.047 | 1.342e+05 | 0.000 | 1 |
| Eggs per clutch | 1.049 | 7.121e-03 | 6.803 | 1.02e-11*** |
| Dolores | 1.229 | 1.342e+05 | 0.000 | 1 |
| Pedregal | 3.477 | 1.342e+05 | 0.000 | 1 |
| Requena | 3.242 | 1.342e+05 | 0.000 | 1 |
| San Martín | 7.530 | 1.342e+05 | 0.000 | 1 |
| Teotihuacán | 0.862 | 1.788e+05 | 0.000 | 1 |
| Texcoco | 2.450 | 1.342e+05 | 0.000 | 1 |
| Tlaxiaca | 1.802 | 1.342e+05 | 0.000 | 1 |
| Toluca | 3.031 | 1.342e+05 | 0.000 | 1 |
| Tzintzuntzán | 2.763 | 1.342e+05 | 0.000 | 1 |
| Valsequillo | 3.115 | 1.342e+05 | 0.000 | 1 |
| **Null deviance:** 1287.51 on 410 degrees of freedom | | | | |
| **Residual deviance:** 426.51 on 399 degrees of freedom | | | | |
| **AIC:** 2103 | | | | |
