# Supplementary material for: Searching for a common host: parasitoids of Lema daturaphila on Datura stramonium in Central Mexico
Source: PeerJ. 2025 Feb 3;13:e18675. doi: 10.7717/peerj.18675 (PMC11801200; doi:10.7717/peerj.18675)
Supplement: Supplemental Information 9 — Estimated values about the number of emerged parasitoid flies were calculated with a Poisson generalized linear model and back-transformed to the original measure scale. The model explains 89.71% of the variance for 2018 and 79.02% of the variance for 2019. [file peerj-13-18675-s009.docx]

|  | **2018** | | | |
| --- | --- | --- | --- | --- |
|  | **Estimate** | **Std. Error** | **Z value** | **Pr(>\|z\|)** |
| Intercept | 1.303 | 0.339 | 0.781 | 0.435 |
| Number of larvae | 1.040 | 0.006 | 6.639 | <0.0001*** |
| **Null deviance:** 55.49 on 7 degrees of freedom | | | | |
| **Residual deviance:** 5.7062 on 6 degrees of freedom | | | |  |
| **AIC:** 33.177 | | | |  |
|  | **2019** | | | |
|  | **Estimate** | **Std. Error** | **Z value** | **Pr(>\|z\|)** |
| Intercept | 2.662 | 0.194 | 5.028 | <0.0001*** |
| Number of larvae | 1.023 | 0.001 | 15.448 | <0.0001*** |
| **Null deviance:** 407.924 on 10 degrees of freedom | | | | |
| **Residual deviance:** 85.552 on 9 degrees of freedom | | | | |
| **AIC:** 135.01 | | | | |
